# Clinical and radiographic efficacy of subtalar screw arthroereisis in the treatment of pediatric flexible flatfoot

**DOI:** 10.1007/s00590-026-04669-2

**Published:** 2026-02-19

**Authors:** Farouk Khury, Emilie Danto, Rita Taurman, Martin Faschingbauer

**Affiliations:** 1 The Ruth and Bruce Rappaport Faculty of Medicine, Technion – Israel Institute of Technology, Haifa, Israel; 2 Division of Orthopedic Surgery, Rambam Health Care Campus, Haifa, Israel; 3 Department of Orthopedic Surgery, University of Ulm, Ulm, Germany; 4 Department of Gynaecology and Obstetrics, Stauferklinikum, Mutlangen, Germany; 5 Department of Orthopedics, Klinik Penzing, Wiener Gesundheitsverbund, Vienna, Austria

**Keywords:** Flatfoot arthroereisis, Pediatric flexible flatfoot, Subtalar arthroereisis, Subtalar screw arthroereisis, SSA

## Abstract

**Background:**

Subtalar arthroereisis has been reported to be an effective treatment technique for flexible flatfoot (FF) in children. Although many devices for this procedure exist, arthroereisis using screws is still globally used. Therefore, this study aims to revisit subtalar screw arthroereisis (SSA) and investigate its outcomes.

**Methods:**

We retrospectively reviewed 353 flexible flatfeet in 178 pediatric patients who underwent SSA between 2007 and 2020. Clinical and radiological assessments were conducted pre-implantation and pre-explantation. Radiographic angles were measured to quantify correction. Statistical analyses included *chi*-squared tests and Student’s t-tests to evaluate clinical improvement and the impact of variables on outcomes.

**Results:**

The mean patient age at implantation was 11.96 years. 96.31% of feet showed clinical improvement postoperatively. Radiographic analysis demonstrated significant correction in most (83.33%) angular measurements, with the calcaneal pitch showing the strongest effect size. Postoperative complications occurred in 41.08% of FF, predominantly pain, and were mainly (84.13%) resolved with non-surgical treatment. 4.25% required implant revision, which was significantly more frequent in the younger age and female group.

**Conclusion:**

SSA for treatment of FF in children showed favorable results regarding improved clinical aspects and radiographic measurements. Nevertheless, an accurate indication for surgical treatment is necessary.

**Level of evidence:**

Clinical retrospective research—Level III.

**Supplementary Information:**

The online version contains supplementary material available at 10.1007/s00590-026-04669-2.

## Introduction

Pediatric flatfoot is a complex three-dimensional deformity of the foot caused by collapse of medial arch, plantar tilt of talus, and eversion of calcaneus [1, 23]. It is one of the most common pediatric skeletal disorders and a frequent cause for clinical orthopedic consultation [23]. Although the current treatment approach for asymptomatic flexible flatfoot (FF) is “watch-and-wait”, intervention may be required in cases of pain and limited activity. Treatment varies from non-surgical activities, such as shoe modification, non-steroidal anti-inflammatory medications (NSAIDs), physiotherapy, and stretching exercises, addressing comorbidities such as obesity, hypotonia, and ligamentous hyperlaxity, to surgical soft tissue procedures, realignment osteotomies, and subtalar joint non-fusion procedures [12].

Ever since its first description in the literature by Chambers in 1946 [5], subtalar arthroereisis (SA) has been one of the most widely debated minimally invasive procedures for the treatment of symptomatic FF. The controversy is due to the fact that although several studies showed that it can improve pain, deformity, and function in FF, it also carries relatively high rates of complications (notably sinus tarsi pain and implant removal), inconsistent outcomes, poorly defined indications/contraindications (especially age, severity, and whether to use adjunctive procedures), and a lack of high-quality long-term evidence [6, 19, 20, 30]. Despite reports of various SA techniques and implants, the orthopedic principles implemented remain undisputed—correction of the excessive foot pronation, hindfoot valgus, and medial arch height by introducing an implant directly or indirectly into the sinus tarsi. [4, 7–9, 16, 18, 21, 24, 29, 32]. Among these options, the screw remains a widely favored implant due to its simplicity, rigid mechanical reliability, and widespread availability compared to newer bioabsorbable or expandable devices [3, 22]. However, these advantages come with the notable drawback of frequently requiring a second procedure for implant removal [22, 30].

While several clinical, radiological, kinematical, biomechanical, and pedobarographic reports have been published [4, 7–9, 16, 18, 21, 24, 29, 32], the exact clinical and radiological outcomes of pediatric FF treated with subtalar screw arthroereisis (SSA) remain unclear. Therefore, this study, which included a high volume of pediatric patients with FF, was conducted to describe the outcomes following SSA, by answering the following questions: 1. What are the outcomes following SSA? 2. Does SSA lead to a clinical improvement of pediatric FF? 3. Can SSA assist in radiological correction of pediatric FF? 4. Which factors correlate to the development of postoperative complications?.

## Methods

### Cohort

#### Study design

This study is a retrospective review of pediatric patients treated for FF with the SSA technique in our institution between 2007 and 2020.

The course of care was as follows: 1. Baseline visit: patients were initially seen, clinically and radiologically examined, and diagnosed with FF on based on their clinical and radiological evaluation in our ambulatory outpatient clinic. As part of the preoperative assessment, all patients had a documented history of symptoms and underwent standardized non-operative management, including activity modification, supportive footwear, stretching/physiotherapy, and orthotic insoles. In cases where non-operative treatment yielded no pain relief and no significant radiological improvement within three months, surgical treatment using SSA was then offered after the exhaustion of conservative measures. 2. Implantation: patients had undergone SSA. 3. First post-implantation follow-up: clinical and radiological examination six weeks following implantation. 4. Second post-implantation follow-up: clinical and radiological examination before implant removal. 5. Explantation: patients had undergone removal of screw either due to reaching skeletal maturity or a complication. 6. Post-explantation follow-up: clinical examination six weeks following implant removal.

The mean interval between pre-implantation and pre-explantation radiographs was 49.76 months (range 4 to 135 months).

Postoperative complications were defined as postoperative sensory deficits, pain (following traumatic injury or without traumatic injury), wound healing disorders (defined as delayed healing or dehiscence, excluding surgical site infections), peroneal contracture or spasm, and fractures.

#### Patient selection criteria and surgical indication

Included in this study were children with remaining skeletal growth, idiopathic symptomatic FF, were not previously operated, had no neurogenic or neuromuscular pathologies including rigid FF, had complete pre- and postoperative clinical and radiological documentations, and had undergone two surgeries: implantation during skeletal growth, and explantation following skeletal growth arrest, of a subtalar arthroereisis screw in our institution. Skeletal growth arrest was assessed using radiographs to evaluate physeal closure, with consideration of the patient’s chronological age and expected skeletal maturity [27].

Diagnosis of idiopathic flexible flatfoot was established based on a combination of clinical and radiographic findings. Clinically, patients presented with a collapsed medial longitudinal arch, hindfoot valgus, and forefoot abduction that corrected with a heel raise (Jack’s Test [13, 17]). Radiographically, lateral and dorsoplantar weight-bearing radiographs demonstrated characteristic angular deviations including a Meary’s angle greater than 4°, a calcaneal pitch less than 20°, a Costa-Bartani angle greater than 125°, a talar declination angle greater than 30°, a talocalcaneal angle often greater than 35°, and a talometatarsal angle often greater than 20°. Surgical intervention with SSA was indicated for symptomatic flexible flatfoot after exhaustion of conservative measures (such as shoe modification, non-steroidal anti-inflammatory medications, physiotherapy, and stretching exercises) and in cases of pain and limited activity.

**Fig. 1:**
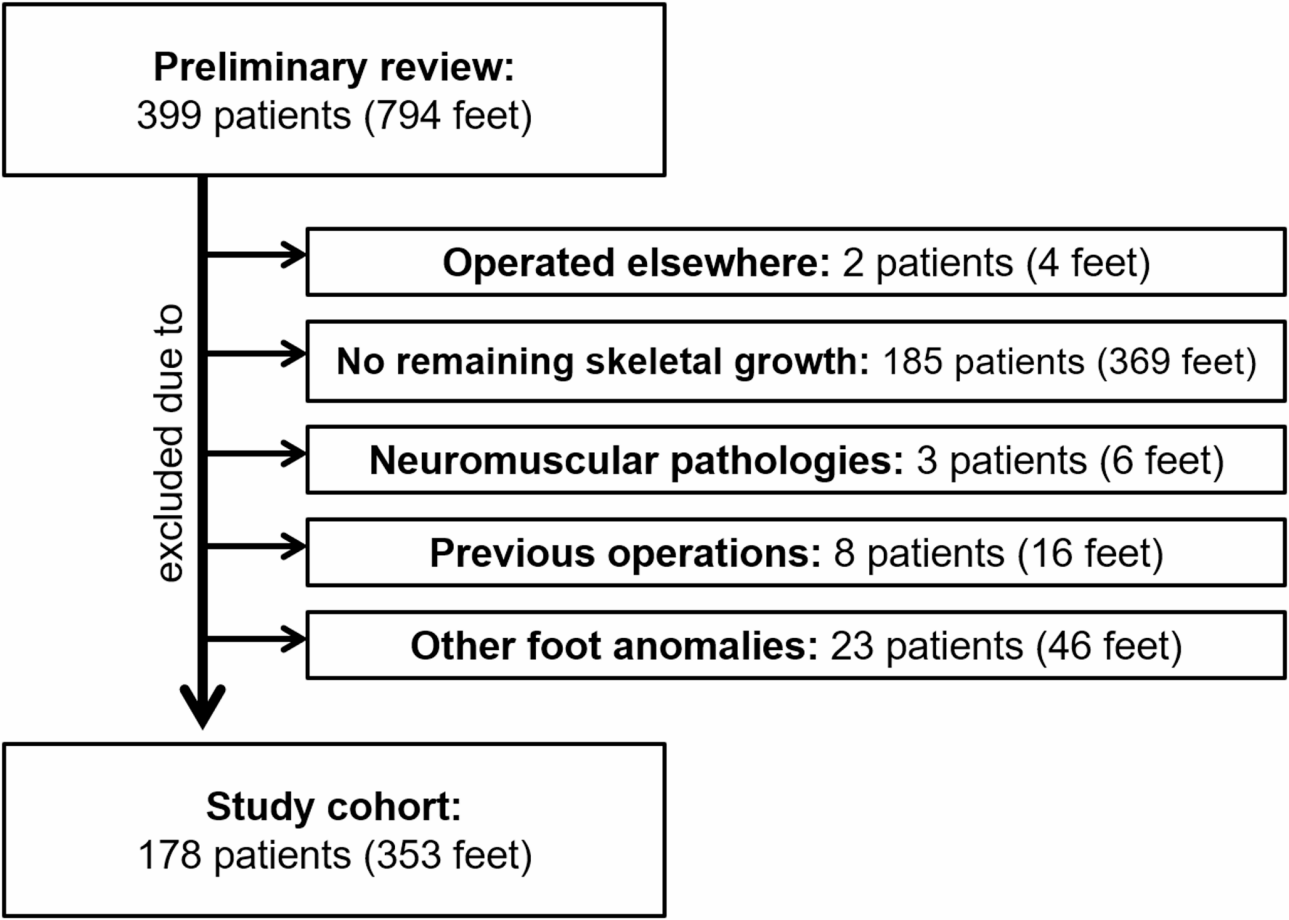
Flow diagram presenting the investigated patient cohort

Primary database query yielded the data of 399 potential patients (794 feet) treated with SSA: two patients (four feet) were excluded due to being operated elsewhere, 185 patients (369 feet) were excluded because they had no remaining skeletal growth, reflecting skeletal maturity, three patients (six feet) were excluded due to neuromuscular disorders, eight patients (16 feet) were excluded due to previous operations, and 23 patients (46 feet) were excluded due to other foot anomalies. Following the exclusion process, 178 patients (353 feet) met the inclusion criteria and were eligible for analysis (Fig. 1).

### Subtalar screw arthroereisis

#### Implant

Patients were implanted with either cannulated or non-cannulated stainless steel cancellous screw with available lengths ranging from 25 to 40 mm and diameter of 6 or 6.5 mm.

#### Operative technique

All surgeries were performed by two experienced attending surgeons in the pediatric orthopedics unit. Information regarding the operative technique is available in supplemental file.

#### Postoperative care

Immediate postoperative full weight-bearing, as tolerated, was allowed. Skin sutures were removed on postoperative day 14. Sport activities were forbidden for six weeks. Patients were presented for an ambulatory clinical and radiological follow-up six weeks following the surgery. Clinical improvement was established once pain and discomfort were reduced, the excessive foot pronation and hindfoot valgus were corrected, and the medial arch was improved. In the absence of a validated patient-reported outcome instrument, ‘clinical improvement’ was defined as a composite of subjective symptom relief and objective physical signs assessed by a pediatric foot and ankle specialist with over 20 years of experience. Specifically, improvement was identified by: (1) restoration of the medial longitudinal arch, (2) correction of hindfoot valgus to a neutral or slight varus position, and (3) a neutral forefoot without abduction.

### Radiographs

#### Protocols

Information available in supplemental file.

#### Angular measurements

**Fig. 2:**
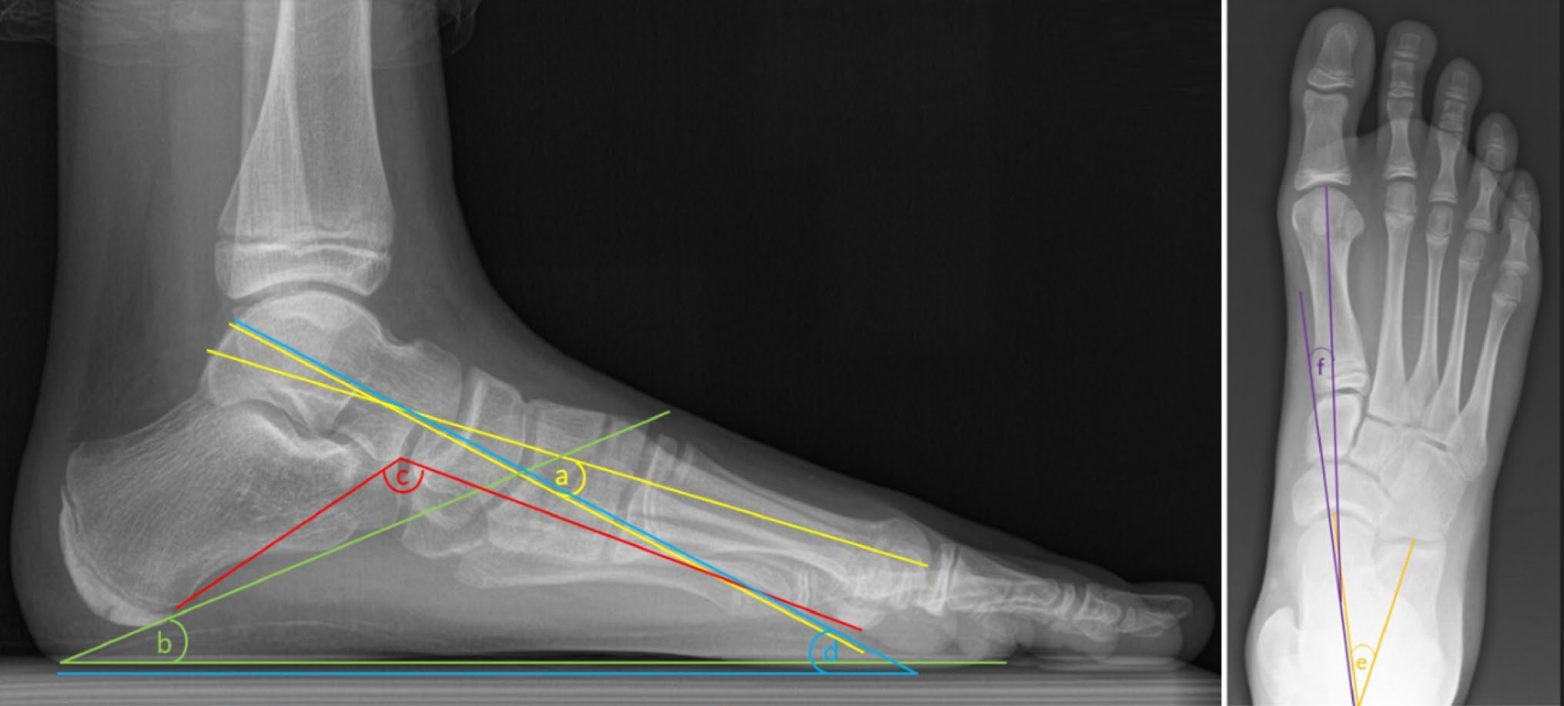
Angular measurements on lateral (left) and dorsoplantar (right) views. a, Meary’s angle; b, calcaneal pitch; c, Costa-Bartani angle; d, talar declination angle; e, talocalcaneal angle; f, talometatarsal angle

To assess the degree of correction, six different angles (four lateral, and two dorsoplantar) were measured on the pre-implantation and pre-explantation radiographs by two of the authors (intra-class correlation coefficient (ICC) = 0.98) using the Centricity™ Universal Viewer (version 6.0, GE Healthcare): Meary’s angle (MA) (normal range: 0° ± 4°), calcaneal pitch (CP) (normal range: 20°–30°), Costa-Bartani angle (CB) (normal range: 120°–125°), and talar declination angle (TD) (normal range: 20°–30°) measured on lateral views, and talocalcaneal angle (TC) (normal range: 15°–35°), and talometatarsal angle (TMT) (normal range: 0°–20°) measured on dorsoplantar views (Fig. 2). The raters were blinded to the time-point (pre-implantation vs. pre-explantation) of the radiographs to minimize bias.

### Statistical analyses

Our institution’s electronic medical records system was utilized to collect patient demographics (age, sex, body mass index (BMI)), preoperative factors regarding treatment and pain, data of the SSA, and radiographic images. Quality metrics consisted of postoperative clinical and radiographic outcomes. Other than descriptive statistics, the statistical model consisted of two primary tests: 1) *chi*-squared (x^2^) test of independence to assess the differences between categorical variables (sex, BMI group, age group, worn-out footwear status, shortened calf muscles status, tripping status, previous treatment status) and clinical improvement (improvement/no improvement) as well as postoperative complication (complication/no complication), and 2) Student’s t-test (or Analysis of Variance (ANOVA) where applicable for multiple groups) to evaluate the change in continuous variables (angular measurements) effect on clinical improvement. Due to the retrospective nature of the study, detailed severity grading (e.g., using a validated scale) and exact duration of each complication were not consistently available for all cases. However, ‘resolved’ was defined as patient-reported improvement and being pain-free following the specified non-surgical treatments. Results were reported with 95% confidence intervals (CI) to indicate the precision of estimates. In cases where a significant *p*-value was calculated, Cramer’s V (for categorical) and Cohen’s d (for continuous parameters) were reported to demonstrate the effect size of the finding: ≤ 0.1 (V) and ≤ 0.2 (d) (weak association), 0.1–0.3 (V) and 0.2–0.6 (d) (moderate association), ≥ 0.5 (V) ≥ 0.6 (d) (strong association) [26]. Given that 175 out of 178 patients underwent bilateral SSA, we acknowledge the potential for within-patient correlation. While the descriptive statistics are presented at the foot level, statistical significance for inferential analyses (*chi*-squared and Student’s t-tests) was interpreted with this potential for non-independence in mind. The significance level was set at *p* < 0.05, and Bonferroni correction was implemented for adjusting *p*-values due to the increased risk of a type I error when making multiple statistical analyses. While Bonferroni correction was applied to mitigate the risk of Type I errors from multiple comparisons, we acknowledge that multivariate models could offer a more comprehensive adjustment for confounding variables such as age, sex, BMI, and follow-up time. This will be considered in future prospective studies. All statistical analyses were performed using IBM SPSS software version 23 (IBM Corporation, Armonk, New York, United States). All variables included in the analyses had complete data, with no missing values.

## Results

### Patient demographics and primary outcomes

The data on 178 patients (353 feet) (168 (47.59%) females, and 185 (52.41%) males) who underwent SSA between December 2007 and July 2018 were meticulously analyzed. The mean follow-up time was 51.47 ± 20.21 (4–135) months. 175 patients had bilateral SSA, 141 of which were operated on in the same setting, and 34 were operated on at another date. Preoperatively, 30 (8.50%) feet had worn-out footwear. Shortened calf muscles were in 22 (6.65%) feet, and tripping was observed in 6 (1.70%) feet. Pain was mainly localized medially (28.61%). 177 (51.14%) feet had specific previous non-surgical treatment; of which, 161 (90.96%) were treated with foot insoles, and 16 (9.04%) with physiotherapy (Table 1).

**Table 1:** Overview of patient demographics

Variable	Cohort (353 feet, 178 patients)
Mean age at implantation ± SD (range), years Age groups in years, n (%) [5–10] [10–12] [12–15]	11.96 ± 1.46 (5.67–14.75) 33 (9.34) 134 (37.96) 186 (52.69)
Sex, n (%) Female Male	168 (47.59) 185 (52.41)
Mean BMI ± SD (range), kg/m^2^	19.02 ± 3.23 (12.82–32.89)
BMI category, n (%) Underweight Normal weight Overweight Obese	12 (3.40) 238 (67.42) 79 (22.38) 24 (6.80)
Preoperative factors, n (%) Worn-out footwear Shortened calf muscles Tripping Previous treatment Insoles Physiotherapy	30 (8.50) 22 (6.65) 6 (1.70) 177 (50.14) 161 (90.96) 16 (9.04)
Preoperative pain location, n (%) Medial Unspecific Ankle Lateral Foot sole Midfoot Sinus tarsi Achilles tendon 5th ray 1st ray Posterior tibial	101 (28.61) 68 (19.26) 22 (6.23) 13 (3.68) 10 (2.83) 10 (2.83) 10 (2.83) 8 (2.26) 4 (1.13) 2 (0.57) 1 (0.28)

N, Number of patients; %, Relative frequency of the entire cohort; SD, Standard deviation; BMI, Body mass index

At the time of implantation, the mean age was 11.96 ± 1.46 (5.67–14.75) years, and the mean BMI was 19.02 ± 3.23 (12.82–32.89) kg/m^2^. The majority of the patients (67.42%) had normal BMI values, 22.38% were overweight, 6.80% were obese, and 3.40% were underweight. The mean length of the implantation surgery per foot was 17.26 ± 6.32 (7.5–69.5) minutes. 99.44% of the implantation surgeries did not have any complications. One foot had an intraoperative complication of a broken K-wire, which was left in situ, and another foot required extension of the surgical incision. Following the implantation surgery, patients were hospitalized for a period between two and eight days, with a mean of three days. It is essential to highlight that several patients were admitted one day before surgery. The implant was removed 49.76 ± 20.37 months following SSA, without any reported complications. At the time of the removal, patients’ age was 16.15 ± 1.4 (10.42–19.76) years (Table 2).

**Table 2:** Overview of surgical features

Variable	Cohort (353 feet, 178 patients)
Mean duration of surgery per foot ± SD, minutes	17.26 ± 6.32 (7.5–69.5)
Intraoperative complications, n (%) Broken K-wire Extension of surgical incision	2 (0.56) 1 (0.28) 1 (0.28)
Mean length of stay (range), days^¥^	3 (2–8)
Interval to implant removal ± SD, months	49.76 ± 20.37
Mean age at explantation ± SD (range), years	16.15 ± 1.4 (10.42–19.76)
Mean follow-up time ± SD (range), months	51.47 ± 20.21 (4–135)

N, Number of patients; %, Relative frequency of the entire cohort; SD, Standard deviation; ^¥^, Includes admission one day before surgery

### Clinical improvement

At the first postoperative outpatient visit, 340 (96.31%) feet were found to have clinician-reported improvement as reduction in pain and discomfort was reported, foot pronation and hindfoot valgus were corrected, and the medial arch was improved. Statistical analysis demonstrated that the younger age group [5–10] years exhibited a weak, yet significant clinical improvement when compared to the older groups (96.96% (95% CI 94.2–98.7) vs. 96.26% (95% CI 94.0–97.5) and 96.23% (95% CI 94.1–97.4), *p* < 0.01, V = 0.089) (Table 3). Patient’s sex, BMI, worn-out footwear, shortened calf muscles, tripping, and previous treatment status demonstrated no significant effect on the clinical improvement.

**Table 3:** Analysis using *chi*-squared (x^2^) test of independence to assess the differences between patient variables and clinical improvement

Variable	Clinical improvement (n = 340), n (%%)	Significance	Cramer’s V	Effect size
Sex, n (%) Female, 168 (47.59) Male, 185 (52.41)	159 (94.64) 181 (97.83)	n.s		
BMI, n (%) Underweight, 12 (3.40) Normal, 238 (67.42) Overweight, 79 (22.38) Obese, 24 (6.80)	10 (83.33) 235 (98.73) 72 (91.14) 23 (95.83)	n.s n.s n.s n.s		
Age at time of implantation (years), n (%) [5–10], 33 (9.34) [10–12], 134 (37.96) [12–15], 186 (52.69)	32 (96.96) 129 (96.26) 179 (96.23)	**	0.089	weak
Worn-out footwear, 30 (8.50)	12 (40.0)	n.s		
Shortened calf muscles, 22 (6.65)	10 (45.45)	n.s		
Tripping, 6 (1.70)	5 (83.33)	n.s		
Previous treatment, 177 (50.14)	108 (61.01)	n.s		

N, Number; %, Relative frequency of the entire cohort; %%, Relative frequency of the patients that were clinically improved; **, *p* < 0.01; *, *p* < 0.05; n.s., *p* > 0.05

### Radiographic correction

#### Meary’s angle (MA)

Prior to SSA, the mean MA was 21.77° ± 8.19° (1°–50°). Following the explantation of the implant, the values were reduced to 14.74° ± 8.22° (0°–40°), closer to the normal range of 0° ± 4°, with a difference of −7.1° ± 7.6°.

#### Calcaneal pitch (CP)

The measurement values of CP increased by 2.1° ± 3.14°, from 15.75° ± 4.33° (0°–32°) to 17.92° ± 4.76° (4°–32°) following explantation, approaching the normal range of 20°–30°.

#### Costa-Bartani angle (CB)

CB decreased by 9.31° ± 6.35°, from 136.83° ± 7.78° (112°–159°) to near normal values (120°–125°) 127.57° ± 7.65° (109°–155°) following implant removal.

#### Talar declination angle (TD)

TD decreased by 3.97° ± 6.19°, from 36.52° ± 6.53° (9°–58°) to 32.62° ± 6.26° (14°–51°) after implant explantation, approaching normal range of 20°–30°.

#### Talocalcaneal angle (TC)

TC was reduced by 2.48° ± 7.39°, from 28.73° ± 6.52° (6°–50°) to 26.19° ± 7.46° (2°–47°) following implant removal, within normal range of 15°–35°.

#### Talometatarsal angle (TMT)

TMT was decreased by 2.81° ± 7.99°, from 13.65° ± 7.38° (0°–40°) to 10.65° ± 7.57° (0°–39°) after explantation, remaining within normal range of 0°–20°.

**Fig. 3:**
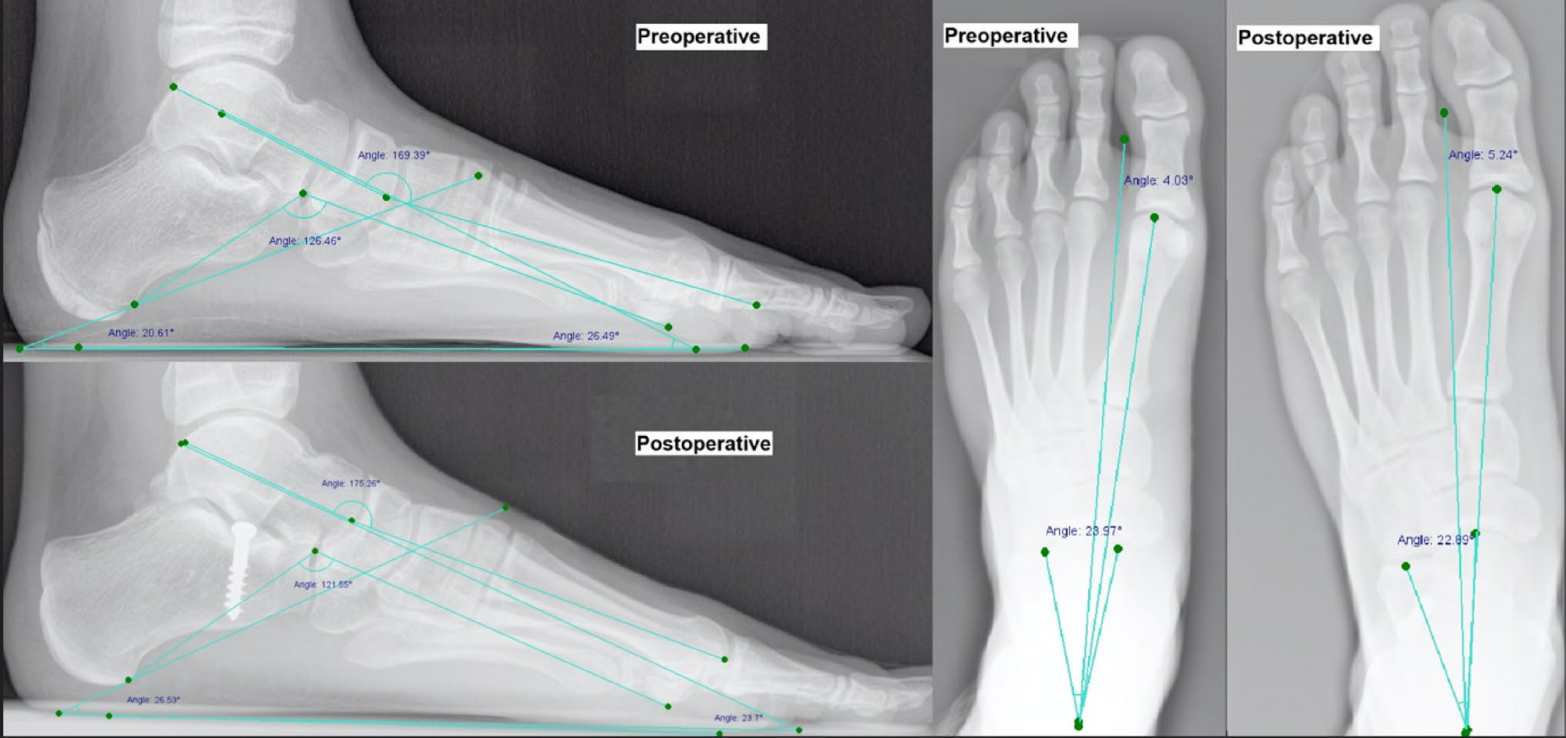
Pre- and postoperative lateral (left) and dorsoplantar (right) angular measurements

Following treatment with SSA and after implant removal, five of the six (83.33%) measured angles demonstrated values approaching the normal range. Furthermore, all angular measurements except TD demonstrated medium-to-strong (d = 0.628–0.915) effect sizes for the entire patient group using Cohen’s d. CP exhibited the strongest effect size (d = 0.915, 95% CI 0.850–0.980) (Table 4). Figure 3 demonstrates the radiographic angular correction following SSA.

**Table 4:** Pre-implantation and post-explantation radiographic angular measurements, including ANOVA for determination of the effect size of the angular measurements

Angle	Normal range (°)	Min (°)	Max (°)	Mean (°)	SD (°)	Significance (*p*-value)	Cohen’s d	Effect size
Pre-implantation in lateral view								
MA	0 ± 4	1	50	21.77	8.19			
CP	20–30	0	32	15.75	4.33			
CB	120–125	112	159	136.83	7.78			
TD	20–30	9	58	36.52	6.53			
Pre-implantation in dorsoplantar view								
TC	15–35	6	50	28.73	6.52			
TMT	0–20	0	40	13.65	7.38			
Post-explantation in lateral view								
MA	0 ± 4	0	40	14.74	8.22			
CP	20–30	4	32	17.92	4.76			
CB	120–125	109	155	127.57	7.65			
TD	20–30	14	51	32.62	6.26			
Post-explantation in dorsoplantar view								
TC	15–35	2	47	26.19	7.46			
TMT	0-20	0	39	10.65	7.57			
Difference between measurements at both visits in lateral view								
MA		−35	20	-7.10	7.60	**	0.628	medium
CP		−11	13	2.10	3.14	**	0.915	strong
CB		−40	16	-9.31	6.35	**	0.824	strong
TD		−24	18	-3.97	6.19	n.s		
Difference between measurements at both visits in dorsoplantar view								
TC		−26	17	−2.48	7.39	**	0.811	strong
TMT		−21	36	−2.81	7.99	**	0.803	strong

SD, standard deviation; Max, Maximum; Min, Minimum; MA, Meary’s angle; CP, calcaneal pitch; CB, Costa-Bartani angle; TD, Talar declination angle; TC, talocalcaneal angle; TMT, talometatarsal angle; **, *p* < 0.01; *, *p* < 0.05; n.s., *p* > 0.05

### Postoperative complications

At the first postoperative ambulatory follow-up, 41.08% of the cohort demonstrated complications. The vast majority of these were due to postoperative pain of some extent (33.14%) (Table 5). This presented mainly without any traumatic injury (30.31%). Although the precise etiology was not consistently documented, the clinical presentation was suggestive of mechanical irritation or local inflammation. Due to the predominance of bilateral cases (175 out of 178 patients), a statistical comparison of pain levels between unilateral and bilateral procedures was not feasible. 2.83% of the operated feet had postoperative pain following a traumatic injury. Ten feet (2.83%) exhibited some sensory deficit (1.98% hypesthesia, 0.56% paresthesia, and 0.28% dysesthesia). 2.55% and 2.27% had peroneal contracture or spasm and wound healing disorders, respectively. Only one foot suffered from a subfibular talus exostosis fracture (0.28%). These complications were treated with watchful waiting, physiotherapy, foot insoles, partial weight-bearing, antibiotics, implant removal, or revision (Table 5). Following this treatment regimen, 84.13% of the patients reported improvement and being pain-free.

**Table 5:** Postoperative complications, treatments, and revisions

Variable	Cohort (353 feet, 178 patients)
Postoperative complication, n (%) Sensory deficit Dysesthesia Paresthesia Hypesthesia Pain Following traumatic injury Without traumatic injury Wound healing disorders Peroneal contracture or spasm Fractures	145 (41.08) 10 (2.83) 1 (0.28) 2 (0.56) 7 (1.98) 117 (33.14) 10 (2.83) 107 (30.31) 8 (2.27) 9 (2.55) 1 (0.28)
Improvement of complications following ambulatory treatment, n (%)	122 (84.13)
Implant revision, n (%) Cause: loss of correction Age: 5–10 years Age 10–12 years Age: 12–15 years Cause: persistent pain Cause: mechanical irritation	15 (4.25) 7 (1.98) 4 (1.13) 2 (0.56) 1 (0.28) 7 (1.98) 1 (0.28)

N, Number of patients; %, Relative frequency

Although 84.13% of the complications were resolved following ambulatory non-surgical treatment, 15 feet (4.25% of the entire cohort) necessitated implant revision due to loss of correction (1.98%) in the younger age group [5–10] years (1.13%), persistent pain (1.98%) and mechanical irritation (0.28%). A total of 4.25% of the entire cohort required implant revision. Due to the retrospective study design, a Kaplan–Meier or cumulative incidence analysis for revision rates was not feasible.

Analysis using *chi*-squared (x^2^) test of independence demonstrated a weakly correlated, yet significantly higher incidence of complications in females than males (60.11% (95% CI 54.0–66.2) vs. 23.78% (95% CI 18.0–29.5), *p* < 0.05, V = 0.081). Furthermore, patients of the younger age group [5–10] years are more significantly inclined to postoperative complications compared to the older groups (72.72% (95% CI 64.5–80.9) vs. 35.82% (95% CI 28.5–43.1) and 39.24% (95% CI 32.5–45.9), *p* < 0.01, V = 0.096). There were no significant differences between the BMI groups regarding the complication rate. Worn-out footwear, shortened calf muscles, and tripping did not contribute to postoperative complications (Table 6).

**Table 6:** Analysis using the *chi*-squared (x^2^) test of independence to assess the differences between patient variables and postoperative complications

Variable	Postoperative complications (n = 145), n (%%)	Significance	Cramer’s V	Effect size
Sex, n (%) Female, 168 (47.59) Male, 185 (52.41)	101 (60.11) 44 (23.78)	*	0.081	weak
BMI, n (%) Underweight, 12 (3.40) Normal, 238 (67.42) Overweight, 79 (22.38) Obese, 24 (6.80)	5 (41.67) 102 (42.85) 24 (30.37) 14 (58.33)	n.s n.s n.s n.s		
Age at time of implantation (years), n (%) [5–10], 33 (9.34) [10–12], 134 (37.96) [12–15], 186 (52.69)	24 (72.72) 48 (35.82) 73 (39.24)	**	0.096	weak
Worn-out footwear, 30 (8.50)	9 (30.0)	n.s		
Shortened calf muscles, 22 (6.65)	9 (40.91)	n.s		
Tripping, 6 (1.70)	0 (0.0)	n.s		

%, Relative frequency of the entire cohort; %%, Relative frequency of the patients who had postoperative complications; **, *p* < 0.01; *, *p* < 0.05; n.s., *p* > 0.05

## Discussion

Characterized by medial arch collapse, plantar tilt of the talus, and eversion of the calcaneus, pediatric FF is one of the most common pediatric skeletal disorders that can cause painful symptoms [1, 23]. Although many variations of the SA technique have been reported [4, 7–9, 16, 18, 21, 24, 29, 32], this study demonstrated that SSA is a minimally invasive and efficient method that leads to significant clinical improvement and radiographic angular correction. Nonetheless, careful patient selection is required to achieve satisfactory outcomes, as several factors in our study proved to have an adverse effect on postoperative progress.

96.31% of the investigated cohort have demonstrated good clinical outcomes at a mean follow-up time of approximately 4.25 years. Even though similar satisfactory rates of 91.48–95% were published in previous studies [2, 7, 25, 28], the methodologies applied differ greatly. While some studies depended on the radiological outcomes, others focused on clinical or subjective improvement. [2, 7, 28, 29, 32] This study combined both the clinical and radiological aspects of the postoperative progress at different time points, providing a comprehensive understanding of the outcomes and potential complications associated with the procedure. Despite the merits of this combined approach, it may not fully capture the variations, as research in FF continues to be limited by the absence of a standardized, universal scoring system for assessment of patient subjective (dis-)satisfaction. Interestingly enough, only the younger age group correlated with clinical improvement. Even though this observation was weak (V = 0.089), it was significant (*p* < 0.01), which might suggest that early intervention may optimize outcomes, possibly due to greater adaptability of the growing foot to the biomechanical changes introduced by SSA [7, 8, 23]. Nonetheless, the long-term implications of this observation remain unclear and require longitudinal studies in the future.

The extent of the three-dimensional pediatric FF deformity becomes more apparent during weight-bearing, and the dynamic projection of these skeletal abnormalities can be most appropriately assessed using plain radiography, which, in contrast to computed tomography and magnetic resonance imaging, is inexpensive, non-invasive, and fast [10, 11]. Following completion of SSA treatment, five out of the six measured angles demonstrated values approaching the normal range (Table 5). Except for Memeo et al. [21], who reported all four measured angles reaching normal values, this is the only study in the current literature reporting such radiological improvements. Most of the angular changes observed are similar to those reported in previous studies [2, 7, 21, 25]. Nonetheless, this study applied effect sizes that help determine the magnitude of change. Statistical analysis demonstrated that while all angular measurements had medium-to-strong changes, the CP exhibited the strongest effect size (Table 4). This is in contrast to De Pellegrin et al. [7], who reported a rather nonsignificant greater improvement of CB than CP. It is worth noting, however, that the authors evaluated the radiological changes before explantation, demonstrating significant improvement in all measurements. Our results have shown that plain radiographs demonstrate significant skeletal correction of pediatric FF using SSA, despite the dependence on inter-observer reliability [2, 11].

While most of the investigated FF (58.92%) had an unremarkable follow-up, without any complications, 41.08% of the entire cohort demonstrated some postoperative pain, sensory deficit, peroneal contracture, wound healing disorder, or fracture (Table 5). Although the observed complication rate exceeds the commonly reported range of 5–30%, it remains relatively close to rates reported for certain implant types and surgical techniques [3, 7, 22, 32]. Moreover, it is important to note that all patients underwent full weight-bearing on bilaterally operated limbs, a factor that can reasonably contribute to postoperative discomfort. This contrasts with other reports in the literature, where non-weight-bearing or partial weight-bearing protocols were implemented postoperatively, potentially contributing to lower reported complication rates. Consequently, to reduce the incidence of postoperative pain and irritation, future protocols might consider a brief initial period of protected weight-bearing (e.g., casting or walking boot) rather than immediate full weight-bearing, alongside rigorous intraoperative sizing to prevent overcorrection. Regardless, 84.13% of these complications were successfully treated and resolved non-surgically, while 10.34% of the complications reported dissatisfaction following ambulatory treatment and required implant revision due to loss of correction, persistent pain, and mechanical irritation. Interestingly, implant complications were significantly more frequent in females than males (101 vs. 44, *p* < 0.05, V = 0.081). This observation might be attributed to foot structure variations and ligamentous laxity differences during childhood and adolescence [14, 31]. Furthermore, 57.14% of the 4.25% who required implant revision were of the younger age group 5–10 years. Analysis demonstrated that this specific age group was significantly more inclined to postoperative complications compared to the older age groups. Similar observations were reported by De Pellegrin et al. [7], as 77.8% of their investigated cohort who required implant revision were aged 5–9 years. Regardless, contrary to the previous study [7], no osteolysis of the talus lateral process was observed in our study. Although obesity and excessive body weight were previously reported to correlate with worse clinical and radiological outcomes [15, 20], our study demonstrated no significant differences in clinical outcomes and postoperative complications between the BMI categories (Tables 3 and 6). Nonetheless, future studies should investigate the variations in outcomes and complications between different patient demographics.

## Limitations

This study has several limitations. First, the study’s retrospective nature inherently introduces potential biases, including incomplete documentation and variability in follow-up intervals. Second, the inclusion of bilateral feet from the same patients may introduce a degree of non-independence in the data, which could affect the generalizability of some statistical findings. While this was considered during interpretation, future prospective studies could benefit from statistical approaches that explicitly account for within-patient clustering, such as mixed-effects models or generalized estimating equations. Third, although the sample size was relatively large and the follow-up duration extended beyond four years, the lack of a control group limits our ability to compare SSA to alternative treatment modalities directly. Comparative studies are essential to establish the superiority or equivalence of SSA in the broader context of pediatric flatfoot management. Fourth, clinical outcome assessment was primarily based on a combination of symptom resolution and radiographic improvement, without using a validated, standardized scoring system for patient-reported outcomes (such as the American Orthopaedic Foot & Ankle Society Score or Oxford Ankle Foot Questionnaire). The absence of these established tools limits the direct comparability of our results with other prospective studies. This limitation reflects a broader issue in pediatric orthopedic literature, where subjective assessment tools are not universally applied or validated for flatfoot interventions. Fifth, while radiographs were used to evaluate angular correction, they offer limited insight into the foot’s dynamic function and soft tissue adaptations. Advanced imaging, such as weight-bearing CT or 3D gait analysis, could provide a more comprehensive understanding of biomechanical changes postoperatively, but is not routinely used due to cost and accessibility. Sixth, some group analyses (e.g., age-related outcomes or sex-based complication rates) were statistically significant but had weak effect sizes (e.g., V = 0.081–0.089), suggesting that while these trends may be relevant, they should be interpreted with caution and require validation in prospective studies with stratified sampling and multivariate analysis. Seventh, although obesity has been implicated in worse postoperative outcomes in previous literature [15, 20], the absence of significant differences between BMI groups in our cohort may be influenced by sample distribution, and larger studies are necessary to confirm these findings. Eighth, while complications were reported, standardized severity grading, the precise duration for each complication and the time to symptom resolution were not consistently available, limiting a more granular analysis of their impact. Future studies should incorporate validated tools for complication assessment. Lastly, the interpretation of our results must consider the influence of our institution’s specific postoperative protocol (e.g., immediate full weight-bearing), which may limit direct comparability with studies employing different rehabilitation strategies [5, 7].

In summary, while this study offers valuable insights into the efficacy of SSA for pediatric flatfoot, future research should focus on prospective, multicenter trials with standardized outcome measures and comparative groups to establish more definitive treatment guidelines.

## Conclusions

In conclusion, this retrospective analysis of a relatively large sample size describes SSA as a minimally invasive surgical technique associated with significant clinical improvement and radiographic correction across multiple angular parameters. While an overall complication rate of 41.08% was observed, the majority of these complications were minor and successfully managed non-surgically, contributing to overall patient satisfaction. Interestingly, younger age at the time of surgery was associated with a statistically significant, albeit weak, correlation with more favorable clinical outcomes, suggesting a potential benefit of earlier intervention that warrants further investigation. This study contributes to the existing literature by providing a large-volume, comprehensive analysis of both clinical and radiographic outcomes of SSA in pediatric flexible flatfoot over an extended follow-up period, specifically evaluating effect sizes for radiographic changes and detailing the spectrum and management of postoperative complications, including factors correlating with their development.

## Supplementary Information

Below is the link to the electronic supplementary material.

Supplementary Material 1

Supplementary Material 2

Supplementary Material 3

## Data Availability

No datasets were generated or analysed during the current study.
