# Supplementary material for: Clinical and radiographic efficacy of subtalar screw arthroereisis in the treatment of pediatric flexible flatfoot
Source: Eur J Orthop Surg Traumatol. 2026 Feb 19;36(1):106. doi: 10.1007/s00590-026-04669-2 (PMC12920407; doi:10.1007/s00590-026-04669-2)
Supplement: Supplementary file 1 — Supplementary Material 1 [file 590_2026_4669_MOESM1_ESM.docx]

*Supplemental Table 5*

Postoperative complications, treatments, and revisions. N, Number of patients; %, Relative frequency.

| **Variable** | **Cohort**  **(353 feet, 178 patients)** |
| --- | --- |
| **Postoperative complication**, n (%)  Sensory deficit  Dysesthesia  Paresthesia  Hypesthesia  Pain  Following traumatic injury  Without traumatic injury  Wound healing disorders  Peroneal contracture or spasm  Fractures | 145 (41.07)  10 (2.83)  1 (0.28)  2 (0.56)  7 (1.98)  117 (33.14)  10 (2.83)  107 (30.31)  8 (2.27)  9 (2.55)  1 (0.28) |
| **Improvement of complications following ambulatory treatment**, n (%) | 122 (84.13) |
| **Implant revision**, n (%)  Cause: loss of correction  Age: 5 – 10 years  Age 10 – 12 years  Age: 12 – 15 years  Cause: persistent pain  Cause: mechanical irritation | 15 (4.25)  7 (1.98)  4 (1.13)  2 (0.56)  1 (0.28)  7 (1.98)  1 (0.28) |

*Supplemental Table 6*

Analysis using the *chi*-squared (x^2^) test of independence to assess the differences between patient variables and postoperative complications. %, Relative frequency of the entire cohort; %%, Relative frequency of the patients who had postoperative complications; **, p < 0.01; *, p < 0.05; n.s., p > 0.05.

| **Variable** | **Postoperative complications**  **(n = 145), n (%%)** | **Significance** | **Cramer’s V** | **Effect size** |
| --- | --- | --- | --- | --- |
| **Sex**, n (%)  Female, 168 (47.59)  Male, 185 (52.41) | 101 (60.11)  44 (23.78) | * | 0.081 | weak |
| **BMI**, n (%)  Underweight, 12 (3.40)  Normal, 238 (67.42)  Overweight, 79 (22.38)  Obese, 24 (6.80) | 5 (41.67)  102 (42.85)  24 (30.37)  14 (58.33) | n.s.  n.s.  n.s.  n.s. |  |  |
| **Age at time of implantation** (years), n (%)  [5 – 10], 33 (9.34)  [10 – 12], 134 (37.96)  [12 – 15], 186 (52.69) | 24 (72.72)  48 (35.82)  73 (39.24) | ** | 0.096 | weak |
| **Worn-out footwear**, 30 (8.50) | 9 (30.0) | n.s. |  |  |
| **Shortened calf muscles**, 22 (6.65) | 9 (40.91) | n.s. |  |  |
| **Tripping**, 6 (1.70) | 0 (0.0) | n.s. |  |  |
