# Supplementary material for: Clinical and radiographic efficacy of subtalar screw arthroereisis in the treatment of pediatric flexible flatfoot
Source: Eur J Orthop Surg Traumatol. 2026 Feb 19;36(1):106. doi: 10.1007/s00590-026-04669-2 (PMC12920407; doi:10.1007/s00590-026-04669-2)
Supplement: Supplementary file 2 — Supplementary Material 2 [file 590_2026_4669_MOESM2_ESM.docx]

**Supplemental**

**Protocols**

Included patients had undergone full radiological studies consisting of lateral (obtained by placing the tube at a distance of around 100 cm from the foot, centered at the base of metatarsals, parallel to the horizontal axis, with the plantar surface of the foot perpendicular to the image receptor) and dorsoplantar (obtained by placing the foot on the detector with the lower limb perpendicular to the floor, and the tube at a distance of around 100 cm centered at the base of the third metatarsal, angled 20 degrees toward the heel) radiographs in upright full weight-bearing position.
