# Supplementary material for: Clinical and radiographic efficacy of subtalar screw arthroereisis in the treatment of pediatric flexible flatfoot
Source: Eur J Orthop Surg Traumatol. 2026 Feb 19;36(1):106. doi: 10.1007/s00590-026-04669-2 (PMC12920407; doi:10.1007/s00590-026-04669-2)
Supplement: Supplementary file 3 — Supplementary Material 3 [file 590_2026_4669_MOESM3_ESM.docx]

**Supplemental**

**Operative technique**

Surgeries were performed directly by or under the supervision of an experienced pediatric orthopedic surgeon (RT). The patients were brought to the operating room and placed in the supine position. General or regional anesthesia was applied. The lower extremity was prepped and draped in a sterile fashion. A tourniquet was placed on the thigh and inflated. Following prophylactic antibiotic administration, a curved, along the skin lines, minimally invasive 1.5 cm skin incision was made over the sinus tarsi. After careful deep dissection, the bony surface of the calcaneus was demonstrated. Under fluoroscopy, a 1.6-mm K-wire was placed in the subtalar joint and inserted in the calcaneus during maximal manual inversion of the foot, parallel to the fibula and calcaneocuboidal joint. The K-wire was overdrilled with a 3.2-mm cannulated drill bit till the far cortex of the calcaneus. Following measurement of length, a stainless-steel cancellous screw was implanted at the level of the sinus tarsi, under the talus lateral process. The position of the hindfoot was then observed with respect to the longitudinal axis of the lower limb and examined under eversion of the calcaneus: if undercorrected, the screw was pulled out a few threads from the calcaneus, if overcorrected, the screw was inserted further into the calcaneus. The foot was considered appropriately corrected if both malleoli were seen from a plantar view. The tourniquet was released, and hemostasis was achieved. Subcutaneous and skin sutures were applied. The wound was sterilely dressed, and the lower limb was wrapped in an elastocompressive bandage. Final radiological scans were performed and documented electronically.
